# Dehumanization in organizational settings: some scientific and ethical considerations

**DOI:** 10.3389/fnhum.2014.00748

**Published:** 2014-09-24

**Authors:** Kalina Christoff

**Affiliations:** Department of Psychology, University of British ColumbiaVancouver, BC, Canada

**Keywords:** dehumanization, empathy, problem solving, reasoning, beliefs, ethics, medicine, decision making

## Abstract

Dehumanizing attitudes and behaviors frequently occur in organizational settings and are often viewed as an acceptable, and even necessary, strategy for pursuing personal and organizational goals. Here I examine a number of commonly held beliefs about dehumanization and argue that there is relatively little support for them in light of the evidence emerging from social psychological and neuroscientific research. Contrary to the commonly held belief that everyday forms of dehumanization are innocent and inconsequential, the evidence shows profoundly negative consequences for both victims and perpetrators. As well, the belief that suppressing empathy automatically leads to improved problem solving is not supported by the evidence. The more general belief that empathy interferes with problem solving receives partial support, but only in the case of mechanistic problem solving. Overall, I question the usefulness of dehumanization in organizational settings and argue that it can be replaced by superior strategies that are ethically more acceptable and do not entail the severely negative consequences associated with dehumanization.

## Introduction

Dehumanizing attitudes and behaviors frequently occur in organizational settings and are often viewed as an acceptable, and even necessary, strategy for pursuing personal and organizational goals. Behind this view, there lie a number of commonly held beliefs about dehumanization. These beliefs are culturally determined, rather than based on scientific observation. One such belief is that subtle forms of dehumanization, such as disrespect, condescension, and neglect, are innocent and inconsequential. It is also commonly believed that empathy interferes with problem solving and that therefore, suppressing our naturally occurring empathy, and the dehumanization this suppression entails, are necessary to help us make better decisions and improve our problem solving capacity.

Are those beliefs supported by the scientific evidence? Here I review social psychological and neuroscientific advances on dehumanization and show that a number of our beliefs about dehumanization are not supported by the evidence. Although the belief that empathy interferes with problem solving is partially supported, the scientific evidence on this is very new and still contentious. Overall, I question the usefulness of dehumanization in organizational settings and argue that it can be replaced by superior strategies that are ethically more acceptable and do not entail the severely negative consequences that are often associated with dehumanization.

## Dehumanization as an everyday phenomenon

Early psychological theories viewed dehumanization as an extreme phenomenon, occurring primarily in the context of ethnic or racial intergroup conflict (Kelman, 1976; Staub, 1989; Opotow, 1990). More recently, however, an expanded view of dehumanization has emerged. This expanded view recognizes that dehumanization can occur in interpersonal as well as intergroup contexts, and is not limited to conditions of overt conflict (for review see, Haslam and Loughnan, 2014). Instead, dehumanization appears to be an everyday social phenomenon, rooted in ordinary social-cognitive processes (Haslam, 2006).

How do people dehumanize others? When someone is dehumanized, they are implicitly or explicitly perceived as lacking qualities that are considered to be characteristically human. According to Haslam’s (2006) dual model of dehumanization, there are two forms of dehumanization corresponding to two different forms of humanness being denied. One is an “animalistic” form of dehumanization in which humans are denied qualities that are considered to distinguish them from animals—qualities such as refinement, self-control, intelligence, and rationality. This form of dehumanization is often discussed in the context of ethnicity, race, and related topics such as immigration and genocide (e.g., Kelman, 1976; Chalk and Jonassohn, 1990).

Dehumanization can also take a “mechanistic” form in which humans are likened to objects or automata and are denied qualities such as warmth, emotion, and individuality (Haslam, 2006). Such “mechanistic” dehumanization is more likely to occur in interpersonal interactions and organizational settings. It is frequently discussed in the contexts of technology (Montague and Matson, 1983), medicine (Szasz, 1973; Fink, 1982; Barnard, 2001), and other domains such as sexual objectification (Fredrickson and Roberts, 1997; Nussbaum, 1999) in which people are often perceived as inert or instrumental.

Dehumanization can also range from blatant and severe to subtle and relatively mild (Haslam and Loughnan, 2014). Such relatively mild dehumanizing behaviors can manifest themselves in the form of subtle disrespect, condescension, neglect, social ostracism and other relational slights (Bastian and Haslam, 2011), often only evident in looks, gestures, and tones of voices. These subtle, everyday forms of dehumanization are often viewed as innocent and inconsequential (e.g., Sue et al., 2007). How does this view compare to the scientific evidence?

## The negative consequences of everyday dehumanization

There is overwhelming evidence for the wide-reaching negative consequences of relatively mild dehumanizing attitudes and behaviors. Dehumanizing others leads to increased anti-sociality towards them in the form of increased aggressive behaviors such as bullying (Obermann, 2011) and harassment (Rudman and Mescher, 2012), as well as hostile avoidance behaviors such as social rejection (Martinez et al., 2011). This increased hostility and aggression are accompanied by reduced moral worth attributed to those who are dehumanized (Opotow, 1990; Haslam and Loughnan, 2014) and they are therefore judged less worthy of protection from harm (Gray et al., 2007; Bastian and Haslam, 2011). The perpetrators of such interpersonal maltreatments themselves may experience negative emotions such as guilt and shame (Baumeister et al., 1995; Tangney et al., 1996), which may lead to even stronger dehumanizing attitudes towards their targets in an attempt to downplay their suffering and justify their maltreatment. Such dehumanization in response to guilt has been demonstrated in intergroup contexts (Castano and Giner-Sorolla, 2006). A vicious cycle may emerge, whereby dehumanization promotes maltreatment and aggression, which further promotes dehumanization.

The negative consequences for those who are dehumanized are also striking. Everyday interpersonal maltreatments can leave its victims feeling degraded, invalidated, or demoralized (Hinton, 2004; Sue et al., 2007). There is extensive research into the negative consequences of being denied autonomy (Ryan and Deci, 2000), betrayed (Finkel et al., 2002), humiliated (Miller, 1993), socially excluded (Baumeister and Leary, 1995; Twenge et al., 2007), or not recognized as a person (Honneth, 1992)—all situations that are likely to be experienced as dehumanizing (Bastian and Haslam, 2011).

When people are mechanistically dehumanized by being treated as objects, as means to an end, or as lacking the capacity for feeling, they tend to enter into “cognitive deconstructive” states that are characterized by reduced clarity of thought, emotional numbing, cognitive inflexibility, and an absence of meaningful thought (Twenge et al., 2003; Bastian and Haslam, 2011). Experiencing this form of dehumanization leads to pervasive feelings of sadness and anger. Also dehumanizing are status-reducing interpersonal maltreatments such as condescension, degradation, or being treated as embarrassing, incompetent, unintelligent, or unsophisticated (Vohs et al., 2007), which lead to feelings of guilt and shame (Bastian and Haslam, 2011).

Such dehumanizing maltreatments are likely to have a detrimental effect on psychological wellbeing. According to self-determination theory (Ryan and Deci, 2000), psychological wellbeing requires that the basic psychological needs of autonomy, competence, and relatedness are met. Dehumanizing maltreatments, however subtle, lead to impaired ability to satisfy these needs and may therefore directly contribute to mental illnesses such as depression, anxiety, and stress-related disorders. In short, the scientific evidence does not support the view of everyday dehumanization as an innocent and inconsequential phenomenon; on the contrary, the evidence clearly demonstrates a range of significant negative consequences.

## The relationship between empathy and problem solving

Another commonly held view about dehumanization concerns the relationship between empathy and problem solving. According to this view, there is a trade-off between empathy and problem solving (e.g., Haque and Waytz, 2012) and the two are mutually incompatible; therefore, suppressing empathy is necessary for effective problem solving. To what extent does psychological and neuroscientific research support this view?

Human thinking and problem solving can be said to occur in two distinct domains: the physical domain, which involves reasoning about the mechanical properties of inanimate objects, and the social domain, which involves thinking about the mental states of others (Jack et al., 2012)– a process also known as “mentalizing” (Frith et al., 1991). Psychological and neuroscientific research shows that empathy—or our capacity to recognize other people’s emotions—is not only compatible with problem solving in the social domain, but that it is also crucial for it (Amodio and Frith, 2006; Harris and Fiske, 2006). On the other hand, is there evidence that empathy is incompatible with problem solving in the physical domain?

A distinction between social and physical problem solving has been suggested at the neural level. Social reasoning about the mental states of others is associated with increased recruitment of the brain’s “default” network and reduced recruitment of the so called “task-positive” network; conversely, “mechanistic” reasoning about physical objects appears to be associated with increased recruitment of the “task-positive” network and reduced recruitment of the “default” network (Jack et al., 2012). Although these two networks are involved in multiple processes and the specificity of their function is still under much debate, they appear to be frequently anti-correlated during conditions of “rest” (Fox et al., 2005) and during many standard cognitive tasks (Shulman et al., 1997).

Anti-correlations between the “default” and the “task-positive” networks were originally interpreted to indicate that the two networks function in opposition to each other and are marked by a negative reciprocal relationship (e.g., Fox et al., 2005). More recently however, neuroscientists have realized that the exact nature of the neural relationship between these two networks is much more complex than a simple obligatory negative reciprocity (e.g., Spreng et al., 2010; Boyatzis et al., 2014). Positive correlations or lack of anti-correlations between the two networks have been observed during creative thinking (Ellamil et al., 2011), mind-wandering (Christoff et al., 2009), and naturalistic film viewing (Golland et al., 2007). Furthermore, it has become apparent that reduced recruitment in one network does not necessarily lead to increased recruitment in the other. With specific relevance to dehumanization, reductions in “default” network recruitment have been observed in the absence of change in recruitment of “task-positive” regions (Jack et al., 2013). While this field is new and still growing, the neuroscientific evidence so far does not support the notion that reduced empathy (or dehumanization) automatically and necessarily leads to improved mechanistic reasoning at the cognitive level.

There is some evidence, however, that the social and physical domains may become incompatible at higher levels of reasoning complexity. The process of relational integration, or considering multiple relations simultaneously, characterizes complex forms of reasoning (Halford et al., 2010) and is specifically associated with increased recruitment of rostrolateral prefrontal cortex (RLPFC) during problem solving in both the physical (e.g., Christoff et al., 2001) and social (Raposo et al., 2011) domains. Problem solving in the two domains may, therefore, become incompatible at higher levels of reasoning complexity due to competition for access to the same neural and cognitive resources.

In short, scientific evidence suggests that the distinction between reasoning in the social and physical domains may be crucial for determining the relationship between empathy and problem solving. In the social domain, empathy is not only compatible with problem solving; it is a crucial component of reasoning about other people’s mental states. In the physical domain, on the other hand, there is some suggestive evidence that empathy and mechanistic problem solving may interfere, especially at higher levels of reasoning complexity (see also Dixon et al., 2014). However, the notion that reductions in empathy automatically lead to improved mechanistic problem solving is not supported by the evidence.

## Questioning the usefulness and ethics of dehumanizing strategies

Dehumanization is sometimes presented as both necessary and beneficial. For example, it has been argued that dehumanization and moral disengagement allows physicians to inflict pain on their patients—pain which is sometimes necessary for diagnosis and treatment (Lammers and Stapel, 2011; Haque and Waytz, 2012). This argument has been extended beyond medical contexts, to argue that dehumanization in general helps people in position of power to make “tough” decisions that may cause pain and suffering for others; it helps by allowing such decisions to be made in a more distant, cold, and rational manner (Lammers and Stapel, 2011). It has also been argued that by dehumanizing patients, health care workers can “protect” themselves against “burnout” from the emotional demands of working with suffering patients (Vaes and Muratore, 2013), and that mechanistic dehumanization of patients in the form of “decomposing people and their symptoms into physiological systems and subsystems” is necessary for “higher level” medical problem solving (Haque and Waytz, 2012).

Whether such “functional” dehumanization is a truly beneficial strategy, however, is highly questionable. It is true that physicians sometimes need to inflict pain on their patients through diagnosis and treatment, but if this pain is necessary for the reduction in the patient’s overall suffering, physicians could mentally focus on this overall improvement as a way of coping. Dehumanizing their patients seems, in comparison, a much more negative and, arguably, much more *dys*functional way of coping—especially considering the profoundly negative consequences it can have for the doctor-patient relationship (Benedetti, 2011). Similarly, avoiding burnout in health care workers can be achieved without requiring them to dehumanize their patients; instead, health care workers could be provided with reduced workload and better support. Furthermore, continually having to suppress their naturally occurring empathic response may create an additional form of stress in some health care workers. Alternative forms of emotional regulation (Gross, 1998; Grandey, 2000) may help reduce health care workers’ stress with fewer costs to themselves and their patients. Overall, the argument that dehumanization helps health care workers provide “better care” (Vaes and Muratore, 2013) only makes sense if “care” itself is understood in a dehumanized mechanistic sense.

It is also true that people in position of power sometimes have to make “tough” decisions that may cause pain and suffering for others. The difficulty in such “tough” decisions, however, comes from their moral nature and the ethical dilemmas they present. Moral reasoning and decisions making by definition require that we use our emotions and our experiences of being human—emotional and otherwise. Dehumanizing those about whom we are making a moral decision would of course eliminate the moral elements of the decision making process (and therefore make it “easier” for the decision maker), but it should also raise some serious ethical concerns. A much more constructive and ethically acceptable way to ease the burden of such difficult moral decisions would be to relieve the person in power of the decision making responsibility and to place it where it rightfully belongs: with the person who will bear the greatest consequences of the decision. In medical contexts, this person would be the patient (or the patient’s chosen substitute decision maker). On the rare occasions when a patient is unable to make such decisions and there is no available substitute decision maker, physicians could seek moral support and advice from others and could allow the necessary time and emotional expenditure it takes to respect the moral and ethical nature of medical decision making.

As well, the argument that mechanistic dehumanization, in the sense of reducing patients to their symptoms and body parts, is necessary for medical problem solving rests on an outdated and largely discredited “biomedical” model of disease. The narrow, exclusive focus on anatomical, physiological, and molecular mechanisms within this “biomedical” model has been criticized and rejected in favor of the much broader “biopsychosocial” model of disease and recovery (Engel, 1977; Benedetti, 2011), which requires that psychological and social factors are included alongside biological factors in medical diagnosis and decision making. Within this newer model, dehumanization would be expected to *impair* medical problem solving by causing the relevance of psychological and social factors to be neglected.

Thus, viewing dehumanization as “functional” and beneficial only makes sense within a very narrow and mechanistic context. What appears “functional” within this narrow context, appears clearly dysfunctional from a broader and more humanized perspective. Far from being necessary, dehumanization in medical contexts can be replaced by superior strategies that are ethically much more acceptable and do not entail the negative consequence that become apparent when dehumanization is viewed from a broader perspective.

## Conclusions

Many of our beliefs about the role of dehumanization are based on implicit empirical claims that can be examined in light of the scientific evidence. Here I examined a number of such beliefs and found relatively little support for them. First, contrary to the commonly held belief that everyday forms of dehumanization are innocent and inconsequential, the evidence shows profoundly negative consequences of such milder forms of dehumanization for both victims and perpetrators. Second, the belief that reductions in empathy automatically lead to improved mechanistic problem solving is not supported by the evidence. Third, the belief that empathy is incompatible with problem solving is partially supported by the evidence, but only if “problem solving” is equated with mechanistic reasoning about inanimate objects in the physical domain. If problem solving is instead equated with mentalizing, or social reasoning about other people’s mental states, this belief is contradicted by the evidence which shows that empathy is a necessary and a crucial element of problem solving in the social domain. Overall, there seems to be a need to reassess our beliefs about the role of dehumanization in organizational settings.

Dehumanization in organizational settings is a highly complex phenomenon with far-reaching implications, from individual, to societal, to global environmental levels. Although scientific evidence can be brought to bear in examining the validity of commonly held beliefs in this area, the present analysis also shows that many of those beliefs carry significant moral and ethical implications. Furthermore, those beliefs may also have implicit normative aspects that have remained unexamined so far.

An interesting case of a complex mixture of an empirical claim and an implicit normative statement may be presented by the argument that suppressing empathy is necessary for problem solving in organizational settings. There is empirical evidence in support of this argument, but only if “problem solving” is reduced to problem solving in the physical domain (i.e., mechanistic problem solving about inanimate objects). Therefore, this argument privileges the value of mechanistic problem solving over the value of problem solving in the social domain, thus making an implicit normative statement. In other words, when employees are encouraged to suppress empathy and focus on “getting the job done”, they are also given the message that mechanistic problem solving is more efficient at getting the job done than empathy or mentalizing. Such implicit normative statements may sometimes lie at the basis of what may appear to be empirically-based arguments.

Recognizing the co-existence of empirical and normative bases of our beliefs about dehumanization can help us develop a more effective approach to their critical examination. While the empirical basis of our beliefs, when identified, can be examined in light of findings from scientific research, the normative aspects of our beliefs are beyond the scope of scientific evidence. Instead, they need to be assessed from ethical, philosophical, and legalistic perspectives. Only an integrated approach that brings together these multiple levels of analysis can help us achieve what seems to be an insurmountable and yet a vitally important task: the humanization of our organizations and, ultimately, the re-humanization of our society.

## Conflict of interest statement

The author declares that the research was conducted in the absence of any commercial or financial relationships that could be construed as a potential conflict of interest.
